# Factors affecting outcome in the treatment of streptococcal periprosthetic joint infections: results from a single-centre retrospective cohort study

**DOI:** 10.1007/s00264-020-04722-7

**Published:** 2020-08-27

**Authors:** Octavian Andronic, Yvonne Achermann, Thorsten Jentzsch, Flurin Bearth, Andreas Schweizer, Karl Wieser, Sandro F. Fucentese, Stefan Rahm, Annelies S. Zinkernagel, Patrick O. Zingg

**Affiliations:** 1 Department of Orthopaedics, Balgrist University Hospital, University of Zurich, Forchstrasse 340, 8008 Zurich, Switzerland; 2 Division of Infectious Diseases and Hospital Epidemiology, University Hospital Zurich, University of Zurich, Zurich, Switzerland; 3 University of Zurich, Rämistrasse 71, 8006 Zürich, Switzerland

**Keywords:** Periprosthetic joint infection, PJI, Streptococcus infection, Biofilm, Rifampin

## Abstract

**Purpose:**

To report and analyse factors affecting the outcome of streptococcal periprosthetic joint infections (PJIs).

**Methods:**

A retrospective analysis of consecutive streptococcal PJIs was performed. Musculoskeletal Infection Society 2013 criteria were used. Outcome was compared with a prospective PJI cohort from the same institution.

**Results:**

The most common isolated streptococcal species was *Streptococcus dysgalactiae* (9/22, 41%) among 22 patients included. Surgical treatment consisted of DAIR (debridement, antibiotics, irrigation and retention) in 12 (55%), one-stage revision arthroplasty in one (4%), two-stage revision arthroplasty in eight (37%) and implant removal in one (4%) patient. An infection free-outcome was achieved in 15 cases (68%), whilst seven (32%) patients failed initial revision and relapsed with the same pathogen, from which six were treated with DAIR and one with one-stage revision arthroplasty. No failures were observed in patients who received a two-stage revision. Failure rates did not differ in the cases treated with rifampin (1/5) from those without 6/17 (*p* = 0.55). There was no correlation between the length of antibiotic treatment and relapse (*p* = 0.723). In all failures, a persistent distant infection focus was identified at the time of relapse. Compared with our prospective PJI cohort, relapse rates were significantly higher 32% vs 12% (*p* < 0.05).

**Conclusion:**

No correlation with the use of rifampin or length of antibiotic treatment was found. No failures were observed in patients who received a two-stage revision, which may be the surgical treatment of choice. A distant persisting infection focus could be the reason for PJI relapse with recurrent hematogenous seeding in the joint.

## Introduction

Periprosthetic joint infections (PJIs) pose a significant problem in orthopaedics due to increased morbidity and mortality [1–3]. Streptococci are the second leading cause of infections and account for about 10% of PJIs [4] (incidence varies between 4 and 16% among reports) [5, 6]. Streptococcal infections were thought to be easy to treat due to their broad antimicrobial sensitivity (including penicillin), but the success rates of treatment are contradictory, and all seem to be inferior due to high relapse infection rates (38–46%) [6, 7]. This is thought to occur because of the ability of biofilm production. There is also currently no consensus regarding the role of rifampin addition in streptococcal biofilm infections, as studies reveal controversial findings [6, 8].

A recent prospective study reported statistically significant improvement in successfully treated patients that received long-term antimicrobial therapy for at least six months [9]. As such, treatment protocols for streptococcal PJI are heterogeneous and include different surgical strategies, different selection as well as dosage and duration of antibiotic treatment.

As there is no consensus on the influence of surgical treatment selection, the role of rifampin addition, as well as the length and type of antibiotic treatment, it was the purpose of this retrospective study to look for any possible associated risk factors in streptococcal PJI treatment failures.

## Patients and methods

A retrospective study using the institutional microbiology databases for streptococcal PJIs between February 2011 and February 2019 was performed. Patients were consecutively identified, and data were obtained by extracting the electronic medical records of patients with streptococcal PJI. An ethical approval was obtained, and all patients signed a written informed consent form. The study was carried out in accordance with the World Medical Association Declaration of Helsinki [10].

PJI was defined as a positive diagnosis based on the presence of either 1 major criterion or 3/5 of the minor criteria, according to the MSIS (Musculoskeletal Infection Society) guidelines [11]. Major criteria were considered the following: sinus tract communicating with prosthesis or pathogen isolated by culture from two separate tissue/fluid samples from the affected joint. Minor criteria consisted of the following: elevated ESR (> 30 mm/h) or CRP (> 10 mg/L), elevated synovial WBC (> 3000 cells/μL), elevated synovial PMN (> 80%), purulence in affected joint and pathogen isolation in one culture [11, 12].

Infections were further classified into early and late infections according to the time of PJI onset following last surgical revision: less than three months for early and more than three months for late infections, respectively. In addition, the type of presentation acute (symptoms < three weeks after index surgery) or chronic, as well as the presumed source of infection, was noted. Haematogenous PJI was defined by diagnosis of infection ≥ one month after surgery, acute manifestation after a pain-free period and positive blood cultures or surgical prosthetic site culture and/or evidence of distant infectious focus consistent with the pathogen [5].

Success of treatment was defined as an infection-free outcome which followed the Delphi International Multidisciplinary Consensus guidelines [13]: well-healed scar with no clinical signs of recurrent or new infection, no further revisions for septic reasons after re-implantation, no PJI-related death and no long-term antimicrobial suppression therapy at least 12 months after surgery [13]. Antibiotic suppressive treatment was defined as continued antibiotic treatment of longer than six months post-operatively.

Extracted data was then compared with the results of the prospective PJI cohort from the same institution, which was started in 2018 and included consecutive periprosthetic joint infections with all types of pathogens. The comparison was performed in order to underline the significant differences in the outcome and failure rates between periprosthetic joint infections from a consecutive cohort from the general population and PJIs with streptococcal pathogens only.

For statistical analysis, SPSS Statistics 24.0 software (IBM, Armonk, NY, USA) was used. Student’s *t* test was used to determine any statistical differences between comparable means. The relative risk (RR) and odds ratios were used to determine if independent factors were associated with specific outcomes. A bivariate Pearson correlation model was used to assess correlation.

The probability of infection-free survival and the respective 95% confidence interval (CI) was estimated using the Kaplan-Meier survival method. For image creation and statistical survivorship analysis, GraphPad Prism V8 Software was used. For all performed analysis, the *α* level was set at 0.05, and all *P* values were 2-tailed.

## Results

A total of 22 patients (13 males, 9 females) with streptococcal PJIs were considered eligible and included in the study, involving 11 hip, eight knee, two shoulder and one hand arthroplasties. One patient had a follow-up of less than 12 months and was excluded from the study. Demographic and clinical data including relevant comorbidities with streptococcal PJI included in our study are summarized in Table 1. The median patient age was 68 years (50 to 90) at time of diagnosis, and the median follow-up was 15 months (range 12–83 months). Five PJIs (23%) were classified as early infection (< 3 months after index surgery), and seventeen (77%) were late infections (> 3 months). In seven patients (32%), previous revision surgery had been performed. The presumed route of infection was hematogenous in 21 cases, whilst one was considered as intra-operative acquired at the time of the last surgery of the involved joint. In eleven patients (50%), a comorbidity that may increase the risk for infection could be identified.

**Table 1:** Characteristics of 22 patients with a streptococcal periprosthetic joint infection

Patient characteristics	Number (%) (*n* = 22)
Sex, male (%)/female (%)	9 (59)/13 (41)
Age years, median (range)	68 (range 50–90)
BMI (kg/m^2^), median (range)	28.4 (range 20–39.1)
Follow-up months, mean (range)	25.5 (range 12–83)
Relevant comorbidities	11 (50%)
Diabetes mellitus	4 (18%)
Illicit drug abuse	2 (9%)
Hepatitis C	2 (9%)
GI ulcer	2 (9%)
Psoriasis (active lesions)	2 (9%)
Cirrhosis	1 (4.5%)
Neoplasia (multiple myeloma)	1 (4.5%)
Previous neoplasia (osteosarcoma)	1 (4.5%)
Previous neoplasia (GI tumour)	1 (4.5%)
Joint, *n* (%)	
Hip	11 (50%)
Knee	8 (36%)
Shoulder	2 (9%)
Hand	1 (5%)
Clinical presentation	
With sinus tract	5 (23%)
Duration of symptoms until septic surgery	
Acute (less than 3 weeks of symptoms)	20 (91%)
Chronic	2 (9%)
Time of presentation with suspected infection	
Early (< 3 months from index surgery)	7 (32%)
Late (> 3 months from index surgery)	17 (77%)
Type of source of infection	
a) Hematogenous	21 (95.5%)
b) Intra- or postoperative acquired	1 (4.5%)
Potential source of infection	
Unknown	13 (59%)
Oral cavity	4 (18%)
Urinary tract infection (graft associated)	1 (4.5%)
IV drug usage	1 (4.5%)
Ipsilateral foot ulcer	1 (4.5%)
Open skin lesions (psoriasis)	1 (4.5%)
Intra- or postoperative acquired	1 (4.5%)
Surgical treatment	
DAIR	12 (55%)
One-stage revision	1 (4.5%)
Two-stage revision	8 (36%)
Implant removal	1 (4.5%)
Antibiotic treatment	
Combination with rifampin	5/22 (22.7%)
Total antibiotic duration, days (range)	82.2 (range 38–133)
Duration of intravenous antibiotics, days (range)	27.4 (range 4–54)
Suppressive antibiotic treatment (SAT)*	3 (13.6%)

*PJI* periprosthetic joint infection, *UTI* urinary tract infection, *DM II* diabetes mellitus type 2, *GI* gastrointestinal, *DAIR* debridement, antibiotics, irrigation and retention

*SAT was defined as continued antibiotic treatment of longer than 6 months postoperatively

### Microbiology

The most commonly isolated streptococcal spp. were the following: *Streptococcus. dysgalactiae* in 9/22 (40.9%) and *S. mitis* in 7/22 (31.8%) of cases, followed by *S. anginosus* in three cases. Positive blood cultures as a sign for haematogenous infection were found in seven out of 13 (54%) infection. Antibiotic resistance to either levofloxacin or clindamycin was found in two patients (Table 2).

**Table 2:** Microbiological findings of 22 streptococcal periprosthetic joint infections (PJI) in our study, summarized after synovial and sonication fluid, tissue biopsies, as well as blood cultures

Streptococcal species	*N* = 22 (100%)
*S. dysgalactiae* (group C *Streptococcus*)	9 (41%)
*S. mitis/oralis*	8 (36%)
*S. anginosus*	3 (14%)
*S. agalactiae* (group B *Streptococcus*)	1 (4.5%)
*S. pneumoniae*	1 (4.5%)
Blood cultures, obtained (%)	*N* = 13 (59%)
Positive	7 (54%)
Negative	6 (46%)
Antimicrobial susceptibility	*N* = 15 (68%)
Pansensitive (penicillin, clindamycin, levofloxacin)	13 (87%)
Levofloxacin Resistance *	1 (6.5%)
Erythromycin and/or clindamycin resistance**	2 (13%)

*Nr* number, *UTI* urinary tract infection, *DAIR* debridement, antibiotics, irrigation and retention, *n/a* not available, *SSI* surgical site infection

*In one case with *S. agalactiae*

**In two cases, one *S. agalactiae* and one *S. pneumoniae*

### Treatment

Surgical treatment consisted of DAIR (debridement, antibiotics, irrigation and retention) in 12 (54.5%) patients, one-stage revision arthroplasty in one (4.5%) patient, two-stage revision arthroplasty in eight (36.5%) patients and implant removal in one (4.5%) patient (Table 2). The median antibiotic treatment duration was 83 days (range 38–133) with a median of 27 days for intravenous antibiotic administration (range 4–54). Rifampin was used in five (22.7%) cases: in two out of 11 patients treated with DAIR, in two out of eight patients with two-stage arthroplasty and in the one patient with one-stage exchange of the prosthesis. Antibiotic choice was according to susceptibility testing of the isolated streptococcal species.

### Outcome

**Fig. 1:**
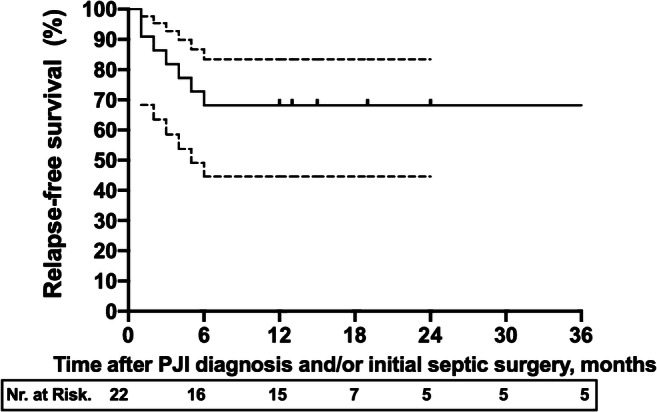
The dotted lines represent the 95% CI (confidence intervals). The relapse-free survival (95% CI) was 68% (53–91%) after 2 years

The survivorship defined as infection free-outcome was achieved in 15 cases (68.1%), whilst seven (31.9%) patients failed initial revision (Fig. 1) and relapsed with the same pathogen. Two out of seven patients relapsed whilst taking antibiotics after DAIR: one at 30 days after PJI surgery and one at 46 days. In six out of seven failures (86%), an initial DAIR surgical treatment was done. One failure occurred after a one-stage revision arthroplasty. No failures were observed in patients who received a two-stage revision.

To treat the relapse, a patient underwent an implant removal and was scheduled for reimplantation but instead remained under suppression therapy due to continuous UTI graft–associated infection. Another two patients underwent two-stage revision arthroplasty and had positive outcomes. There was one patient who relapsed for three consecutive times with the same pathogen (*S. dysgalactiae*) after repeated two-stage revision. He was suffering from ongoing untreated psoriasis skin lesions, which may constitute a distant infectious focus. He was placed on suppression therapy until dermatologic clearance. Three other patients remained under suppression antibiotic treatment due to incompliance in two (untreated dental infection and continued illicit drug abuse), and one patient was not operable (Table 3).

**Table 3:** Characteristics of seven out of 22 periprosthetic joint infections cases with treatment failure

Patient Nr	Time to failure	Surgical treatment	Route of infection/source	Initial isolated microorganism	Isolated microorganism at failure	Rifampin use	Planned antibiotic treatment duration
Patient 1 (hip)	1 month	DAIR	Hematogenous/dental infection	*S. mitis/oralis*	Negative but under antibiotic treatment	No	SAT (due to untreated severe dental infection and increased risk of relapse)
Patient 2 (hip)	3 months	DAIR	Hematogenous (UTI, graft-associated)	*S. anginosus*	*S. anginosus*	No	43 days
Patient 3 (hip)	6 months	One-stage revision	Hematogenous/unknown focus*	*S. dysgalactiae*	*S. dysgalactiae*	Yes	93 days
Patient 4 (hip)	4 months, under SAT	DAIR	Hematogenous/continued illicit drug injections	*S. dysgalactiae*	*S. dysgalactiae*	No	SAT (due to incompliance and continued IV illicit drug abuse)
Patient 5 (knee)	2 months	DAIR	Hematogenous/active gastrointestinal ulcer	*S. dysgalactiae*	Negative but under antibiotic treatment	No	3 months, relapse at 2 months
Patient 6 (knee)	1 month	DAIR	Hematogenous (possibly through active gastrointestinal ulcer)	*S. dysgalactiae*	*S. dysgalactiae*	No	SAT (palliative care due to inoperability for revision arthroplasty)
Patient 7 (hip)	5 months	DAIR	Hematogenous/psoriasis lesions	*S. dysgalactiae*	*S. dysgalactiae*	No	93 days

* Distant focus suspected due to elevated systemic inflammatory parameters and no signs of local infection; Nr number, *UTI* urinary tract infection, *DAIR* debridement, antibiotics, irrigation and retention, *n/a* not available, *SSI* surgical site infection, *SAT* suppressive antibiotic treatment

Failure rates did not statistically differ in the cases treated with rifampin (1/5) from those without 6/17 (relative risk (RR) = 0.56, *p* = 0.55). The Pearson correlation test did not show any correlation between length of antibiotic treatment and relapse risk (*p* = 0.723). One patient with a failure after DAIR was treated for six weeks with antibiotics, a shorter period as compared with other participants (Table 3). The most common organism that was found in the failure group was represented by *S. dysgalactiae*—5/7 (71.4%) of cases. There was an increased risk for this pathogen to lead to a relapse when compared with the rest of pathogens all together (odds ratio = 8.12, *p* = 0.038).

In all failures, a persistent distant infection focus at time of relapse was identified or suspected: one graft infection, two active gastrointestinal ulcers, one with open skin lesions due to psoriasis, one purulent dental infection, one patient with continued drug abuse and repetitive skin infections and one patient with persistent elevated inflammatory markers and no signs of local infection.

**Fig. 2:**
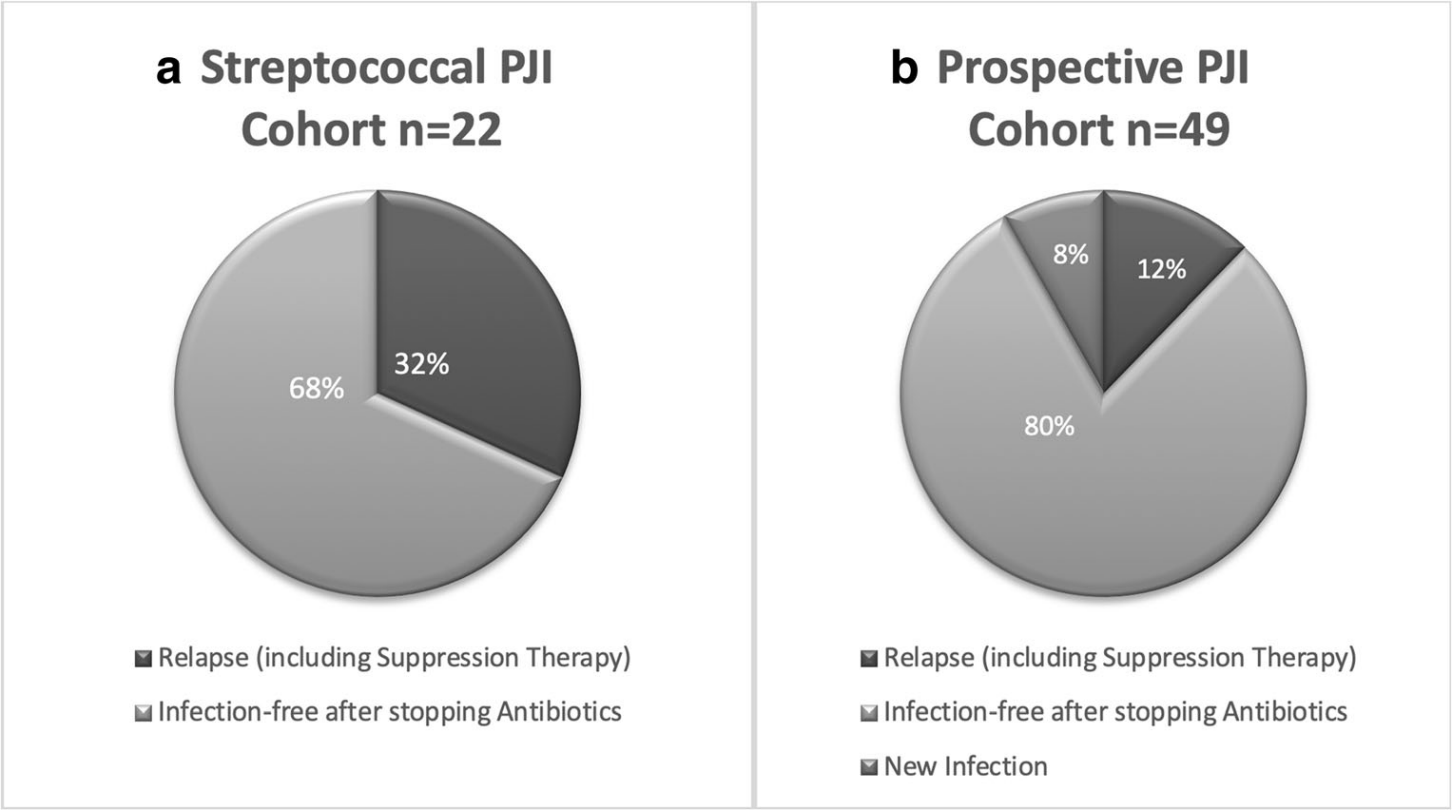
Final outcomes of streptococcal PJIs (**A**) compared with the prospective PJI cohort including all pathogens (**B**). *Pathogens included *Staphylococcus aureus* (8, 16.7%), coagulase-negative staphylococci (14, 29.2%), *Streptococcus* species (6, 12.5%), *Enterococcus* species (2, 4.2%), *Pseudomonas* species (1, 2.1%), *Escherichia coli* (0), other gram-negative rods (4, 8.3%), anaerobes (1, 2.1%), *Cutibacterium* species (4, 8.3%), fungi (1, 2.1%), other monobacterial pathogens (0), culture negative PJI (3, 6.3%) and polymicrobial (4, 8.3%).

When compared with the prospective PJI cohort from our institution (*n* = 49) with a minimum follow-up of 12 months, relapse rates in the streptococcal cohort were significantly higher: 32% vs 12% (p<0.05) (Fig. 2). The main pathogens from the prospective cohort included *S. aureus* in 8 (16.7%), coagulase-negative staphylococci in 14 (29.2%), *Streptococcus* spp. in six (12.5%), *Cutibacterium* spp. in four (8.3%) and Gram-negative rods in four (8.3%) of patients (Fig. 2).

## Discussion

Streptococcal PJIs should be easily be treatable because of their acute presentation making a DAIR possible the good susceptibility to most of our available antibiotics [14, 15]. As such, the streptococcal PJIs are currently not evaluated as “difficult-to-treat” infections. However, there is increasing evidence that these infections should be labeled as “difficult to treat” due to high failure rates [6, 7]. The current study supports these studies, as the infection-free survivorship of treated streptococcal PJI was much lower (68.1%) than in the consecutive prospective cohort from our institution (87.7%).

The role of antibiofilm antibiotics against streptococcal biofilms is still poorly understood. There are reports that demonstrated that group B streptococci are able to produce biofilms [16, 17]. An in vitro study [18] demonstrated the eradication of streptococcal biofilms necessitated (> 125-fold higher) concentrations of all tested antibiotics as compared with the eradication of planktonic bacteria [18]. A recent retrospective study by Renz et al. [9] analysed different antibiotic treatment regimens on the outcome of streptococcal periprosthetic joint infections [9]. The addition of rifampin was not associated with better outcome, which results in accordance with a previous published study from the same institution [6]. On the other hand, there are contradictory reports stating that rifampin addition does achieve better results [7, 8]. In our study, no relationship between rifampin addition and clinical outcome could be found. Further studies are therefore warranted for clarification.

In terms of surgical treatment, previous reports did not observe substantial differences between the types of septic surgery [6–8]. Although there is evidence that DAIR should be surgically sufficient for streptococcal PJIs [19], the recent multicentre study by Lora Tamayo et al. [7] showed a worse prognosis than previously reported in a series of 462 DAIRs. In our cohort, we achieved clinical cure in all cases treated with a two-stage revision arthroplasty. This finding is supported by another recent study by Citak et al. [20], where streptococcal isolation was identified as a risk factor for failure in one-stage revision arthroplasty for PJI.

Previous studies were able to identify a distant infectious focus only in a minority of cases [6, 21, 22], whilst all failures in our study occurred in patients with a potential persistent source of distant infection focus. As such, an active interdisciplinary search to find the source of haematogenous infection should be pursued in all patients to decrease the risk of relapse.

There are limitations of our study. First, the design is not a matched case-control study and is retrospective, although a comparison with a prospective PJI cohort was performed. Second, our study number is low and we deal with a variety of different cases regarding streptococcal species, infection route, prosthesis type and surgical and antimicrobial treatments.

In conclusion, our data of 32% relapse rate supports the need for treating streptococcal PJI as difficult to treat. We did not find any significant correlation with the use of rifampin or length of antibiotic treatment. The most common isolated microorganism among failures was *S. dysgalactiae*. However, no failures were observed in patients who received a two-stage revision. A persistent distal infection focus was often suspected in cases with treatment failure. An aggressive surgical treatment together with appropriate identification and treatment of a distant primary focus should improve outcomes in treatment of streptococcal PJIs.

## Data Availability

Data was stored in a local repository RedCap with access provided to the study staff and principal investigator.
